# Prolactin Monitoring in Adults Prescribed Risperidone at a Tertiary Mental Health Service: Evaluating Current Practices in Al Ain, United Arab Emirates

**DOI:** 10.1192/bjo.2026.11692

**Published:** 2026-06-30

**Authors:** Salma Al Alazeezi, Zahra Ahmed, Duha Al Ali, Mouza AlDhaheri, Syed Fahad Javaid

**Affiliations:** 1 Behavioural Sciences Institute, Al Ain, UAE; 2 Department of Psychiatry, College of Medicine and Health Sciences, United Arab Emirates University, Al Ain, UAE

## Abstract

**Aims:**

Risperidone is associated with a high risk of antipsychotic induced hyperprolactinaemia, and routine biochemical monitoring is recommended. This audit aimed to assess adherence to local hospital guidelines on prolactin monitoring for adult outpatients prescribed risperidone, which align with the National Institute for Health and Clinical Excellence (NICE) Guideline CG178 at the Behavioural Sciences Institute (BSI) in Al Ain,United Arab Emirates, and to identify priorities for improving monitoring and documentation.

**Methods:**

We conducted a retrospective audit of adult patients (≥18 years) initiated on risperidone and followed in BSI outpatient clinics between 2023 and 2025. We reviewed electronic case notes and lab records. The inclusion criteria were at least six months of risperidone treatment and follow-up at BSI. Exclusions included short-term use (<6 months), death, loss to follow-up, or starting risperidone elsewhere. Audit standards were based on Al Ain hospital guidance: (1) baseline prolactin measurement before antipsychotic treatment, and (2) repeat prolactin at 4–6 months. For each patient, we recorded age, sex, diagnosis, and whether prolactin tests were done, with reasons noted if not. Data were analysed as counts and percentages.

**Results:**

Of 240 patients on risperidone during the audit, 159 were excluded (115 treated for less than six months, 24 lost to follow-up, 18 transferred, 2 died), leaving 81 for analysis. The sample included 53 males (65.4%) and 28 females (34.6%), aged 18–94 (mean 45.8). The most common diagnoses were schizophrenia spectrum and other psychotic disorders (55.6%), followed by developmental disorders including autism and intellectual disability (14.8%), major depression (11.1%), neurocognitive disorders (8.6%), bipolar disorder (4.9%), obsessive–compulsive disorder (2.5%), and single cases of adjustment disorder and cerebral palsy (1.2% each). Baseline prolactin was measured in 12 patients (14.8%), 69 (85.2%) had none, with a recorded reason in only one case (1.2%). At 4–6 months, prolactin was checked in 11 patients (13.6%), with reasons documented in 3 (3.7%).

**Conclusion:**

Adherence to local prolactin monitoring for adults on risperidone at BSI was very low, with poor documentation of reasons for missed tests. Given the potential clinical consequences of unrecognised hyperprolactinaemia, these findings highlight an important patient safety gap. Targeted interventions like staff education, electronic reminders, and clearer documentation expectations are needed, along with a re-audit to assess progress.

No financial sponsorship was received for this project.

